# Supplementary material for: Insulin sensitivity is preserved in mice made obese by feeding a high starch diet
Source: eLife. 2022 Nov 17;11:e79250. doi: 10.7554/eLife.79250 (PMC9711519; doi:10.7554/eLife.79250)
Supplement: Figure 3—source data 2. [file elife-79250-fig3-data2.zip › Figure 3 Western Blots source data.pptx]

## Slide 1
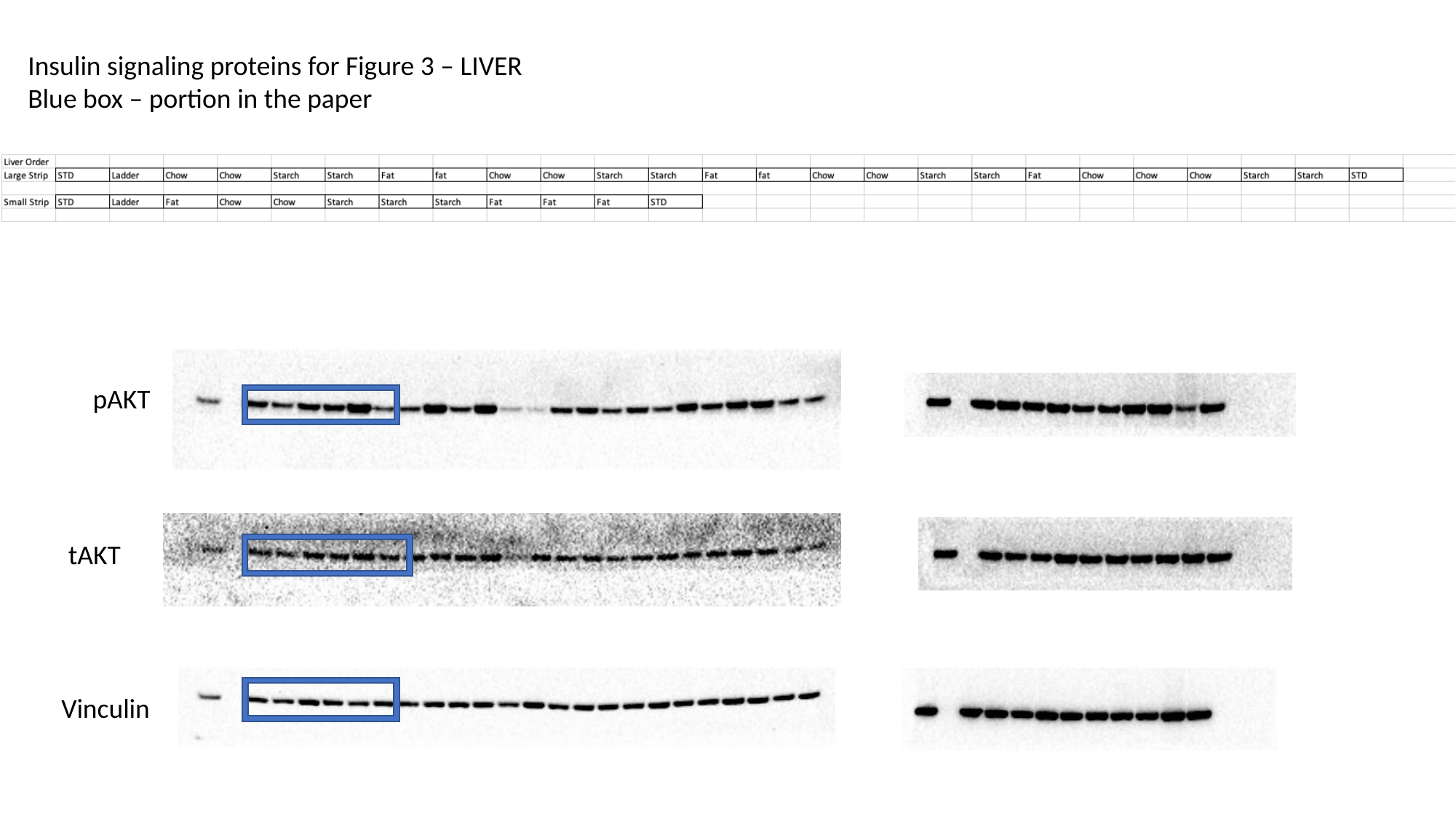

Insulin signaling proteins for Figure 3 – LIVER
Blue box – portion in the paper
pAKT
tAKT
Vinculin

## Slide 2
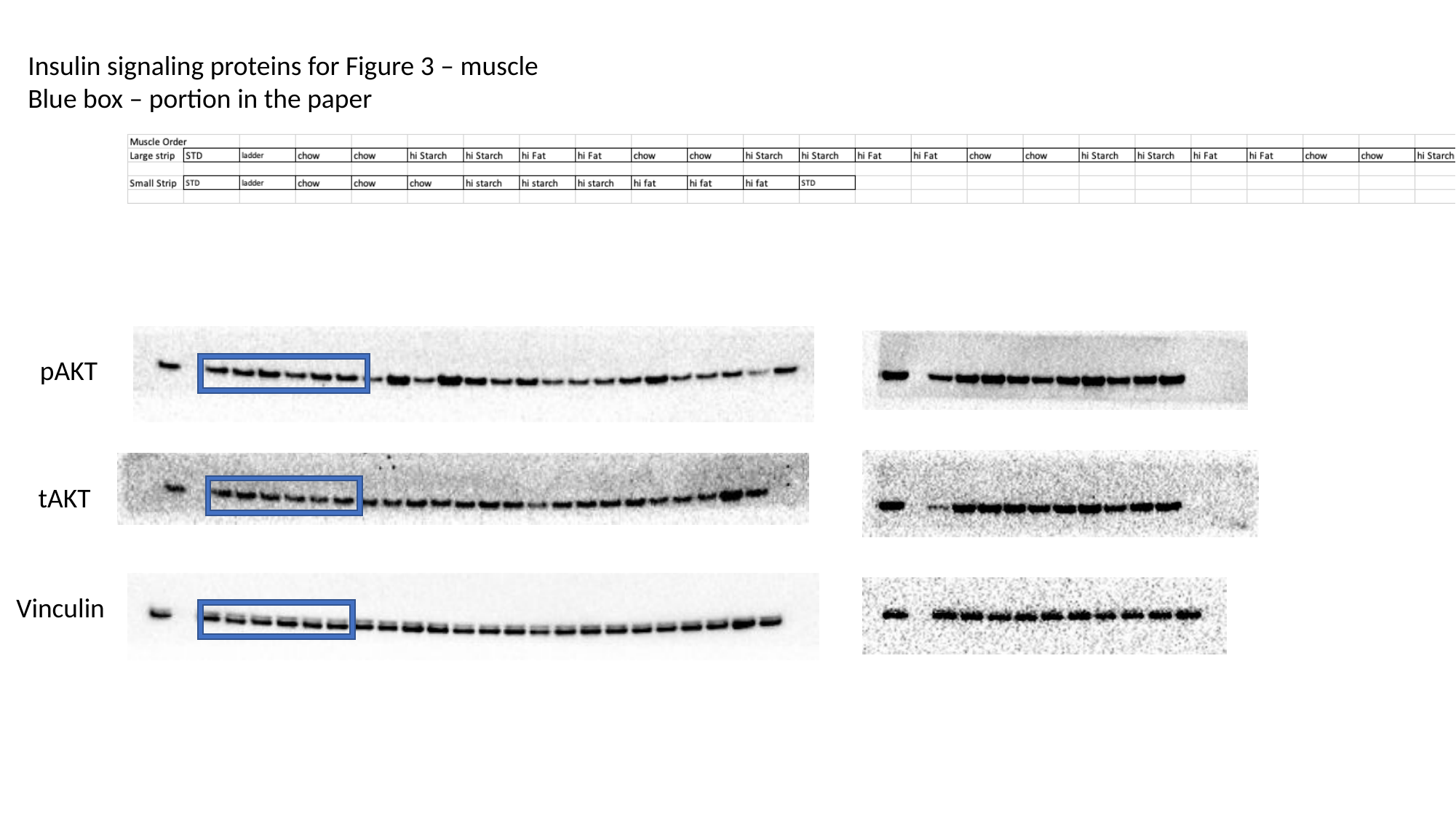

Insulin signaling proteins for Figure 3 – muscle
Blue box – portion in the paper
pAKT
tAKT
Vinculin

## Slide 3
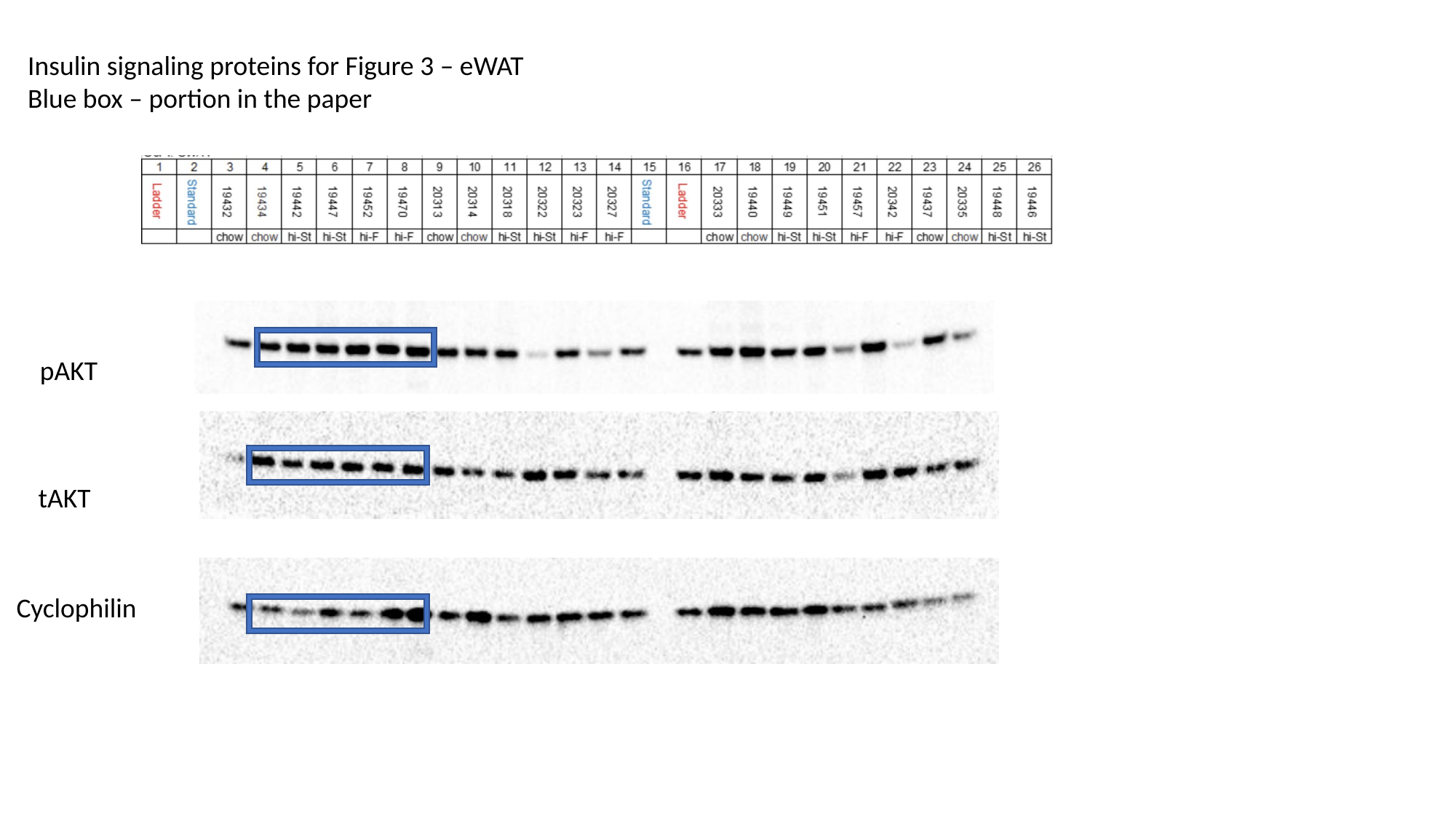

Insulin signaling proteins for Figure 3 – eWAT
Blue box – portion in the paper
pAKT
tAKT
Cyclophilin

## Slide 4
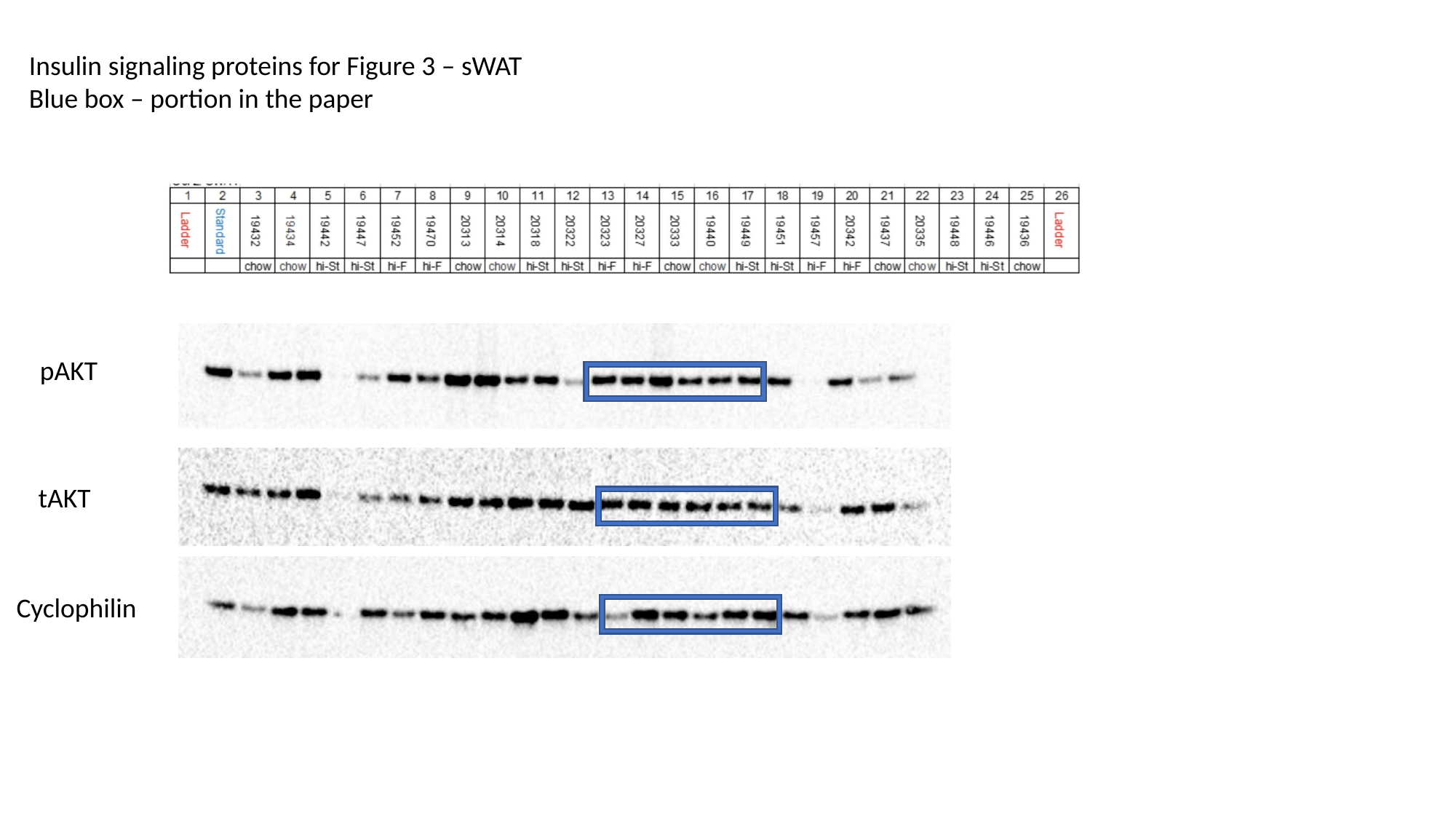

Insulin signaling proteins for Figure 3 – sWAT
Blue box – portion in the paper
pAKT
tAKT
Cyclophilin
