# Supplementary figures and images for: Insulin sensitivity is preserved in mice made obese by feeding a high starch diet
Source: eLife. 2022 Nov 17;11:e79250. doi: 10.7554/eLife.79250 (PMC9711519; doi:10.7554/eLife.79250)

## Slide 1
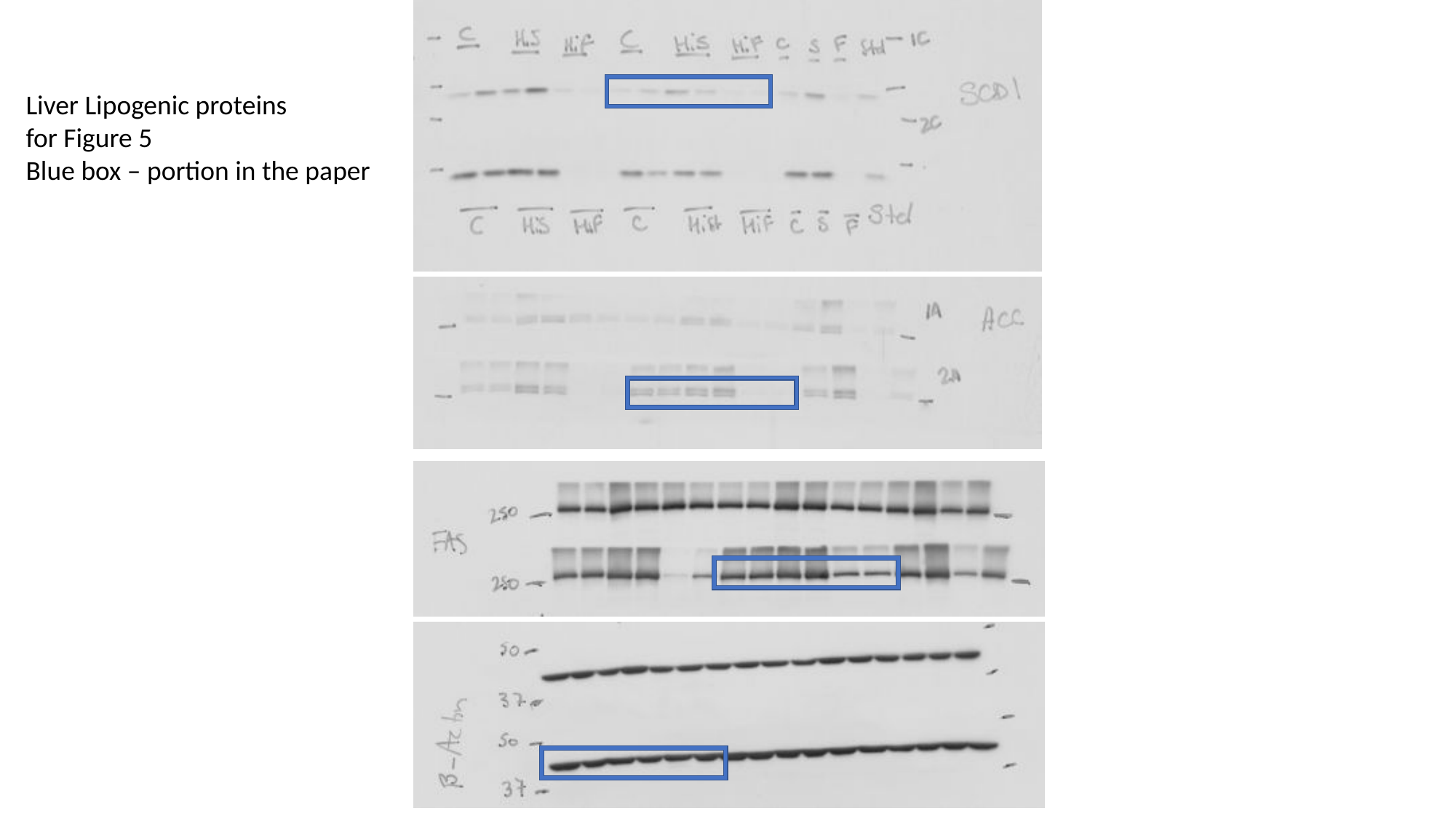

Liver Lipogenic proteins
for Figure 5
Blue box – portion in the paper

Supplement: Figure 5—source data 2. [file elife-79250-fig5-data2.zip › Figure 5 Western Blots source data.pptx]
