# Supplementary material for: Insulin sensitivity is preserved in mice made obese by feeding a high starch diet
Source: eLife. 2022 Nov 17;11:e79250. doi: 10.7554/eLife.79250 (PMC9711519; doi:10.7554/eLife.79250)
Supplement: Supplementary file 1. [file elife-79250-supp1.docx]

**Supplementary File 1a: Muscle Metabolomics**

| Concentration (nmol/g wet weight) | Chow | Hi-ST | Hi-F |
| --- | --- | --- | --- |
| Glucose | 164 ± 38 | 197 ± 49 | 234 ± 43 |
| Lactate | 3517 ± 275 | 4101 ± 244 | 4539 ± 604 |
| E4P | 3357 ± 240 | 2983 ± 282 | 2928 ± 302 |
| Glucose-6-Phosphate | 3167 ± 231 | 2878 ± 257 | 2902 ± 256 |
| glyceradelhyde-3-phosphate | 8.4 ± 1.1 | 26.1 ± 6.5 | 38.1 ± 16.7 |
| R5P | 22.3 ± 4.3 | 38.0 ± 8.6 | 44.4 ± 7.7 |
| S7P | 36.4 ± 4.9 | 38.7 ± 5.2 | 43.7 ± 5.7 |
| Fructose-6-Phosphate | 330 ± 127 | 305 ± 91 | 232 ± 72 |
| Glucose-1-Phosphate | 327 ± 57 | 289 ± 56 | 241 ± 65 |
| dihydroxyacetone-phosphate | 105 ± 9 | 213 ± 43 | 285 ± 94 |
| ribulose-5-phosphate | 66 ± 17 | 131 ± 36 | 135 ± 28 |
| NAD | 2893 ± 136 | 2625 ± 107 | 2249 ± 159* |
| Pyruvate | 326 ± 18 | 430 ± 47 | 436 ± 59 |
| Succinate | 78 ± 9 | 123 ± 12 | 147 ± 24* |
| a-ketoglutarate | 7.0 ± 2 | 3.6 ± 0.6 | 3.0 ± 0.2 |
| Malate | 214 ± 28 | 184 ± 9 | 175 ± 12 |
| UDP-GLC | 14.5 ± 0.9 | 15.5 ± 0.6 | 12.8 ± 0.9 |
| NADP | 115 ± 4 | 118 ± 5 | 117 ± 7 |
| 6-PG | 12.9 ± 1.1 | 18.2 ± 2.5 | 21.0 ± 2.9 |
| Fumarate | 105 ± 15 | 113 ± 19 | 148 ± 29 |
| 3-phosphogycerate | 90 ± 18 | 64 ± 7 | 56 ± 8 |
| FBP | 283 ± 24 | 377 ± 72 | 489 ± 209 |
| NADH | 767 ± 83 | 1296 ± 164 | 1385 ± 306 |
| Citrate | 79 ± 11 | 61 ± 3 | 82 ± 13 |
| phosphoenolpyruvate | 6.0 ± 0.4 | 5.3 ± 0.3 | 5.4 ± 0.4 |
| NADPH | 506 ± 15 | 523 ± 22 | 484 ± 26 |
| Acetyl-CoA | 24.5 ± 1.8 | 24.3 ± 3.5 | 17.3 ± 3.4 |

Data expressed as mean +/- SEM. n = 8-10. *different to chow p<0.05

**Supplementary File 1b: Liver Metabolomics**

| Concentration (nmol/g wet weight) | Chow | Hi-ST | Hi-F |
| --- | --- | --- | --- |
| Glucose | 12115 ± 1075 | 11935 ± 1246 | 9806 ± 1296 |
| Lactate | 4144 ± 312 | 3243 ± 518 | 3062 ± 499 |
| E4P | 377 ± 29 | 371 ± 27 | 298 ± 80 |
| Glucose-6-Phosphate | 397 ± 30 | 391 ± 31 | 333 ± 73 |
| GAP | 8.5 ± 1.0 | 7.1 ± 1.4 | 6.8 ± 1.4 |
| R5P | 612 ± 104 | 723 ± 141 | 589 ± 129 |
| S7P | 3834 ± 439 | 4510 ± 291 | 3679 ± 378 |
| Fructose-6-Phosphate | 24.8 ± 5.3 | 26.1 ± 7.5 | 49.6 ± 16.7 |
| Glucose-1-Phosphate | 32.5 ± 4.8 | 28.9 ± 3.8 | 28.6 ± 7.5 |
| dihydroxyacetone-phosphate | 47.1 ± 3.5 | 38.0 ± 5.9 | 30.5 ± 7.7 |
| ribulose-5-phosphate | 505 ± 120 | 652 ± 109 | 543 ± 110 |
| NAD | 5713 ± 518 | 4967 ± 315 | 5324 ± 704 |
| Pyruvate | 145 ± 14 | 129 ± 19 | 180 ± 25 |
| Succinate | 458 ± 26 | 451 ± 37 | 562 ± 30* |
| a-ketoglutarate | 5.4 ± 1.5 | 6.0 ± 1.9 | 11.6 ± 6.1 |
| Malate | 367 ± 28 | 403 ± 22 | 590 ± 90*# |
| UDP-GLC | 684 ± 79 | 635 ± 49 | 509 ± 93 |
| NADP | 1566 ± 136 | 1457 ± 172 | 1355 ± 145 |
| 6PG | 111 ± 14 | 91 ± 16 | 53 ± 10* |
| Fumarate | 379 ± 33 | 351 ± 59 | 575 ± 93# |
| 3-phosphogycerate | 231 ± 22 | 197 ± 17 | 201 ± 38 |
| FBP | 59 ± 8 | 58 ± 6 | 60 ± 16 |
| NADH | 1890 ± 182 | 1659 ± 224 | 1946 ± 381 |
| Citrate | 102 ± 12 | 130 ± 5 | 139 ± 9* |
| phosphoenolpyruvate | 14.6 ± 1.4 | 12.1 ± 1.1 | 13.5 ± 2.4 |
| NADPH | 1220 ± 77 | 1074 ± 98 | 825 ± 166 |
| Acetyl-CoA | 226 ± 22 | 179 ± 23 | 137 ± 32 |

Data expressed as mean +/- SEM. n = 8-10. *different to chow p<0.05; # different to Hi-ST p<0.05.

**Supplementary File 1c: Diet Composition**

| Diet | Chow | High Starch Diet | | | High Fat Diet | | | | |
| --- | --- | --- | --- | --- | --- | --- | --- | --- | --- |
|  | % Energy | Weight (g) | Energy (kJ) | % Energy | Weight (g) | Energy (kJ) | | % Energy | |
| Casein |  | 175 | 2923 | 16.15 | 175 | 2923 | | 16.22 | |
| Sucrose |  | 100 | 1670 | 9.23 | 100 | 1670 | | 9.27 | |
| Corn starch |  | 500 | 8350 | 46.13 | 100 | 1670 | | 9.27 | |
| Mineral mix |  | 39.2 | 0 | 0.00 | 39.2 | 0 | | 0.00 | |
| Trace minerals |  | 11.4 | 0 | 0.00 | 11.4 | 0 | | 0.00 | |
| Bran |  | 43.4 | 725 | 4.00 | 43.4 | 725 | | 4.02 | |
| Methionine |  | 2.6 | 43 | 0.24 | 2.6 | 43 | | 0.24 | |
| Gelatine |  | 34.8 | 581 | 3.21 | 34.8 | 581 | | 3.23 | |
| Choline bitartrate |  | 3.4 | 0 | 0.00 | 3.4 | 0 | | 0.00 | |
| Safflower oil |  | 26 | 980 | 5.42 | 26 | 980 | | 5.44 | |
| Lard |  | 75 | 2828 | 15.62 | 250 | 9425 | | 52.31 | |
| AIN vitamins |  | 11.4 | 0 | 0.00 | 11.4 | 0 | | 0.00 | |
| Cellulose |  | 100 | 0 | 0.00 | 100 | 0 | | 0.00 | |
| Total ex. water |  | 1122.2 | 18100 | 100 | 897.2 | 18017 | | 100 | |
| Water |  | 800 | 0 | 0.00 | 400 | 0 | | 0.00 | |
| Total inc. water |  | 1922.2 | 18100 | 100 | 1297.2 | 18017 | | 100 | |
| **Macronutrients** | | | | | | | | | |
| Protein | **23** | 234.1 | 3909 | **21.6** | 234.1 | | 3909 | | **21.7** |
| Carbohydrate | **71** | 621.7 | 10382 | **57.4** | 221.7 | | 3702 | | **20.5** |
| Fat | **6** | 101 | 3808 | **21.0** | 276 | | 10405 | | **57.8** |
| Energy Density kJ/g | **13** | **9.42** | | | **13.89** | | | | |
